# *Anaerobutyricum*—An Emerging Butyrate-Producing Genus with Potential Relevance to Host Health

**DOI:** 10.3390/microorganisms14061304

**Published:** 2026-06-10

**Authors:** Chunyu Yan, Mengqing Zhou

**Affiliations:** National Key Laboratory of Pig Genetic Improvement and Germplasm Innovation, Jiangxi Agricultural University, Nanchang 330045, China; 13858516426@163.com

**Keywords:** *Anaerobutyricum*, metabolism, gut microbiota, pigs, next-generation probiotics

## Abstract

*Anaerobutyricum* is a Gram-positive, obligately anaerobic genus within the family Lachnospiraceae that is widely distributed in the gut microbiota of humans and animals. This genus has attracted increasing attention because of its ability to produce short-chain fatty acids, particularly butyrate, a key microbial metabolite involved in intestinal homeostasis, immune regulation, and host energy metabolism. The genus currently comprises only two validly described species, *Anaerobutyricum hallii* and *Anaerobutyricum soehngenii*. Despite this limited taxonomic representation, accumulating evidence has linked variation in *Anaerobutyricum* abundance to host health and disease. In humans, alterations in *Anaerobutyricum* abundance have been linked to metabolic, inflammatory, and neurodegenerative disorders. In livestock, especially pigs, limited evidence suggests that this genus may also be associated with growth-related traits, intestinal health, and reproductive performance. In this review, we summarize current knowledge of the taxonomy, physiological characteristics, genomic features, metabolic potential, and major factors influencing the abundance of *Anaerobutyricum*. We further discuss its reported associations with human health and its possible relevance to animal production, with particular attention to pigs at different developmental stages. Overall, *Anaerobutyricum* appears to be a promising functional genus; however, most available evidence remains association based rather than causal, livestock studies are still sparse, host interaction mechanisms remain poorly understood, and its utility as a probiotic candidate, biomarker, or microbiome-based intervention target requires further strain-level, mechanistic, and in vivo validation.

## 1. Introduction

The mammalian gastrointestinal tract harbors a highly diverse and dynamic microbial ecosystem composed of bacteria, archaea, fungi, protists, and viruses [1]. With advances in high-throughput sequencing technologies and culturomics, the “microbial dark matter” of the gut microbiome has gradually been uncovered [2,3]. The gut microbiome plays essential roles in nutrient metabolism, immune development, the maintenance of epithelial integrity, and host physiological homeostasis. Previous studies have suggested that the phylum Bacillota (formerly Firmicutes) is one of the core phyla of the intestinal microbiota of mammals, and that the family Lachnospiraceae within this phylum is widely distributed and prevalent in the guts of humans and animals [2,4]. Members of Lachnospiraceae are widely recognized for their ability to ferment dietary substrates and produce short-chain fatty acids (SCFAs), particularly butyrate [5,6], which serves as an important energy source for colonocytes and participates in the regulation of immune responses, epithelial barrier function, and host metabolism [7,8,9,10].

As a member of the Lachnospiraceae family, *Anaerobutyricum* is frequently detected in the human gut microbiota in a wide range of studies [11,12,13] and has been suggested to be an important component of the core gut microbiome [14]. This genus has attracted increasing attention because of its potential functional relevance to host health. *Anaerobutyricum* is a Gram-positive, obligately anaerobic genus that currently comprises only two validly described species, *Anaerobutyricum hallii* (*A. hallii*, formerly *Eubacterium hallii*) and *Anaerobutyricum soehngenii* (*A. soehngenii*) [15,16]. Although taxonomically limited, this genus is widely distributed in the gut microbiota of humans and animals and is known for its capacity to utilize a broad range of carbohydrates and to produce butyrate and other metabolites.

Accumulating evidence suggests that *Anaerobutyricum* is associated with multiple physiological and pathological states, particularly metabolic and inflammatory disorders in humans [17,18]. Its abundance can also be influenced by diet, age, physiological status, and disease conditions [19,20,21]. In livestock, especially pigs, only limited studies have reported possible associations between *Anaerobutyricum* abundance and production-related traits, such as growth performance [22,23] and reproductive traits [24]. However, current knowledge remains limited in several respects. First, the genus currently includes only two validly described species. Second, most published studies are descriptive or association based, and causal relationships remain unclear. Third, evidence in livestock is still sparse. In addition, the mechanisms underlying host–*Anaerobutyricum* interactions remain poorly understood, and further genomic, functional, and in vivo studies are needed to evaluate its probiotic potential and practical applicability.

Therefore, this review aims to summarize current knowledge of *Anaerobutyricum*, including its taxonomy, physiological and genomic characteristics, factors affecting its abundance, and reported associations with host health and disease. We also discuss its possible relevance to animal production, particularly in swine, while distinguishing association-based observations from the limited studies providing functional validation, and highlight current knowledge gaps and future research priorities.

## 2. General Characteristics of *Anaerobutyricum*

### 2.1. Taxonomy and Distribution of Anaerobutyricum

*Anaerobutyricum* is a genus within the family Lachnospiraceae that was proposed in 2018 following the taxonomic reclassification of previously described *Eubacterium* species isolated from human fecal samples [16]. Among the two currently recognized members of this genus, *A. hallii* was originally isolated from human fecal samples in 1974 by Holdeman et al. [25] and was first classified as *Eubacterium hallii* because of its numerous phenotypic similarities to members of the genus *Eubacterium* [25,26]. Subsequent 16S rRNA gene phylogenetic analysis indicated that *E. hallii* had been misclassified, and a later polyphasic taxonomic approach led to the establishment of the genus *Anaerobutyricum* within the family Lachnospiraceae [16]. To date, according to the List of Prokaryotic names with Standing in Nomenclature (LPSN), *Anaerobutyricum* comprises two species with validly published names: *A. hallii* and *A. soehngenii. A. hallii* was previously classified as *Eubacterium hallii*, while the type strain of *A. soehngenii* was initially considered part of the *Eubacterium hallii* group before being recognized as a distinct species within the newly established genus [16,27]. Although taxonomically limited, *Anaerobutyricum* is widely distributed in the intestinal tract of mammals. Most reported isolates have originated from human fecal samples, whereas additional isolates have also been described from pigs and monkeys. Collectively, these findings suggest that *Anaerobutyricum* is a widely distributed but still relatively understudied member of the mammalian gut microbiota.

### 2.2. Morphological, Physiological, and Biochemical Characteristics of Anaerobutyricum

Members of the genus *Anaerobutyricum* are obligately anaerobic, Gram-positive, non-motile, non-spore-forming rods that occur singly or in pairs. The optimum growth temperature for both *A. hallii* and *A. soehngenii* is 37 °C, whereas their optimum pH values differ, with values of 7.5 and 6.5, respectively [16]. Members of the genus can utilize a broad range of substrates, including monosaccharides, disaccharides, and sugar alcohols. However, species-specific differences in substrate utilization have been reported. For example, *A. hallii* ferments mannitol and grows well on lactose, whereas *A. soehngenii* lacks these abilities [16]. The major end products of carbohydrate fermentation are butyrate and formate, and members of the genus are negative for oxidase and catalase activity [16]. In addition, *Anaerobutyricum* is characterized by glycerol/diol dehydratase (GDH) activity [15,28]. This metabolic feature may also be linked to interspecies cross-feeding, as genome-scale metabolic modeling suggested that *A. soehngenii* is capable of consuming 1,2-propanediol, whereas various other gut bacterial species may serve as producers of this intermediate [29]. Cellular fatty acid profiles differ between species, with *A. hallii* predominantly containing C14:0, C16:1 cis9, and C18:1 cis11 dimethyl acetal (DMA), whereas *A. soehngenii* is mainly characterized by C16:0, C18:1 cis9, C18:0, and C18:1 cis11 DMA [16]. The genomic DNA G+C content of the genus is approximately 38.2 ± 4.0 mol%, and the type species is *A. hallii* [16].

## 3. Genomic Characteristics of *Anaerobutyricum*

### 3.1. Phylogenomic Classification

Advances in next-generation sequencing have provided novel genomic insights across a broad range of bacterial species [30]. Bioinformatics approaches are widely used for genomic analysis, enabling a shift in research focus from phenotypic assays to genomic prediction and facilitating the integration of genomic and phenotypic data to evaluate probiotic potential [31]. Compared to the extensively studied probiotic *Bifidobacterium* [32,33,34] and lactobacillus [35,36,37] and their rich genomic resources, only a limited number of *Anaerobutyricum* strains have been isolated and characterized, resulting in scarce genomic information for this genus [38]. The genomic analyses summarized here are based on publicly available genome resources, and the included genomes were selected according to their taxonomic assignment to *Anaerobutyricum*, public availability, annotation completeness, and relevance to the comparative analyses presented. However, the currently available genomes remain limited in number and may not fully represent the diversity of the genus. As of 27 December 2025, a total of 26 strains belonging to two recognized *Anaerobutyricum* species were available in the NCBI database, with their general genome features summarized in Table S1. Most *Anaerobutyricum* strains were isolated from human feces, except for strains BSM_383_APC_4H and MSJ_10, which were isolated from pig feces [39] and monkey feces [40], respectively. This indicates that current knowledge of the genomic characteristics of this genus is still primarily based on human-derived strains, and future isolation of more strains from diverse animal hosts is necessary to more comprehensively assess its diversity. The genome sizes range from 3.19 Mb to 3.89 Mb (median = 3.44 Mb), and the number of protein-coding sequences (CDSs) per genome ranges from 2792 to 4432 (median = 3060). The G+C content was relatively conserved, ranging from 37.92% to 38.65% (median = 38.225%). To prevent taxonomic misassignment, all 26 *Anaerobutyricum* strains were taxonomically annotated using GTDB-Tk 2.4.0 based on the Genome Taxonomy Database release 220 [41].

**Figure 1:**
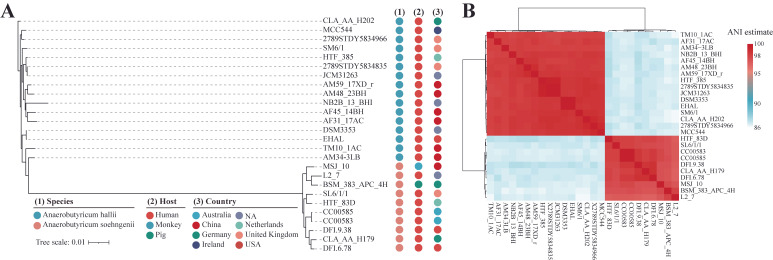
Phylogenetic relationships and genome-level similarity among 26 *Anaerobutyricum* strains. (**A**) Maximum-likelihood phylogenomic tree reconstructed from shared orthologous genes and visualized with iTOL (v7) (http://itol.embl.de). The tree resolves the strains into two main clades corresponding to the two recognized species, *A. hallii* and *A. soehngenii*. Strains from different hosts and geographic origins are distributed within the same clades, indicating no obvious source-specific phylogenetic clustering in the currently available dataset. (**B**) Heatmap of pairwise average nucleotide identity (ANI) values among the 26 genomes. Two high-similarity within-species blocks and lower between-species values are evident, supporting clear species-level separation.

For a better understanding of the genomic diversity of *Anaerobutyricum*, a phylogenomic tree was reconstructed. Orthologous genes shared among the 26 genomes were identified using OrthoFinder (v2.5.4), followed by multiple sequence alignment with MAFFT (v7.520). A maximum-likelihood phylogenomic tree was reconstructed using IQ-TREE (v2.2.6) with the best-fit substitution model automatically selected by ModelFinder and 100 ultrafast bootstrap replicates (as implemented in OrthoFinder with the -T iqtree option). The tree was visualized using iTOL (v7) (Figure 1A). The phylogenetic tree showed that *Anaerobutyricum* strains clustered into two groups, both originating from a common ancestor. More specifically, Figure 1A resolves the 26 genomes into two well-defined clades corresponding to the two recognized species within the genus. Branching patterns within each clade were relatively compact, indicating close relatedness among strains of the same species, whereas the split between the two major clades was markedly deeper, consistent with species-level divergence. Notably, *Anaerobutyricum* strains from different hosts and countries clustered on the same branch, indicating that their phylogenetic relationship was not directly tied to their isolation sources.

Average nucleotide identity (ANI) values were used to measure the nucleotide-level similarity between genomes, with the species boundary cut-off set at 95–96% [42]. Pairwise ANI values were calculated using FastANI (v1.32). The ANI values among the *Anaerobutyricum* genomes ranged from 86.22% to 99.99% (Figure 1B). Specifically, the intra-specific ANI values ranged from 96.70% to 99.99%, while the inter-specific ANI values ranged from 86.22% to 87.38%. This species separation was further supported by the ANI heatmap (Figure 1B), which showed two high-similarity blocks along the diagonal corresponding to within-species comparisons and markedly lower ANI values between the two blocks for between-species comparisons. These results indicate significant genetic differences between the two species within the genus *Anaerobutyricum*, but relatively limited variation among strains belonging to the same species [43]. Together, the phylogenomic tree and ANI matrix consistently support the presence of two genetically coherent species within the currently available *Anaerobutyricum* genomes. A recent comprehensive genomic study analyzed 84 *Anaerobutyricum* genomes, including both isolate genomes and metagenome-assembled genomes (MAGs) [38]. Consistent with our observations, that study reported that *A. hallii* and *A. soehngenii* have longer genomes and lower GC content compared with non-human-derived *Anaerobutyricum* spp., and that both species lack known virulence factors. These findings support the robustness of our genome-based characterization despite differences in dataset composition.

### 3.2. Anaerobutyricum Pan-Genome

**Figure 2:**
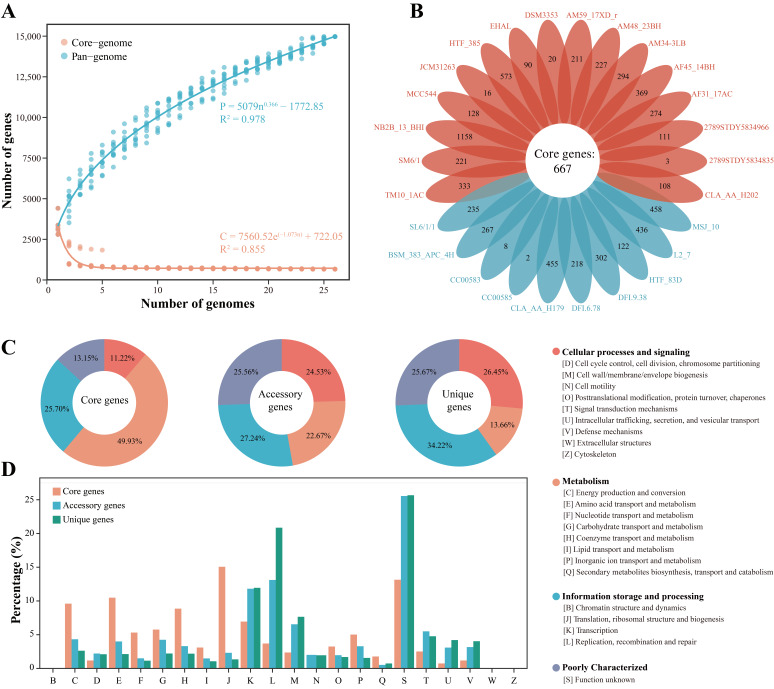
Pan-genome structure and functional composition of 26 *Anaerobutyricum* strains. (**A**) Accumulation curves of the pan-genome (cyan) and core genome (ruby). The continuously increasing pan-genome curve and the shrinking core genome indicate an open pan-genome. (**B**) Distribution of core and unique genes among strains. The relatively small shared core and the wide variation in strain-specific genes illustrate substantial inter-strain genomic heterogeneity. The strain names are colored according to species, where ruby and cyan denote *A. hallii* and *A. soehngenii*, respectively. (**C**) Relative functional composition of COG categories in the core, accessory, and unique genomes. (**D**) Detailed COG category definitions corresponding to panel C. The core genome is enriched in housekeeping functions, whereas the accessory and unique genomes contain larger proportions of genes related to adaptation, regulation, and genome plasticity.

The microbial pan-genome is defined as the collection of all genes that are present in a specific species, comprising the core genome (genes shared by all strains), the accessory genome (genes shared by only some strains), and the unique genome (strain-specific genes) [44]. To assess the genetic diversity within *Anaerobutyricum*, a pan-genome analysis was conducted using the 26 genomes available in the NCBI database. The 26 genomes were annotated with Prokka (v1.14.6) [45] and subjected to pan-genome analysis with Roary (v3.13.0) using a BLASTP (v2.12.0) identity threshold of 95% [46]. The *Anaerobutyricum* pan-genome increased continuously with the addition of every new genome, while the core genome size gradually decreased. The pan-genome curve showed an upward trend, and the fitted curve for the pan-genome profile indicated a positive exponent, suggesting that *Anaerobutyricum* has an open pan-genome (Figure 2A). As shown more explicitly in Figure 2A, the total number of genes in the pan-genome did not approach a clear plateau as additional genomes were added, whereas the core genome progressively declined before stabilizing at a relatively small set of shared genes. This pattern indicates that sequencing additional *Anaerobutyricum* strains would likely reveal further gene diversity. The *Anaerobutyricum* pan-genome comprised 14,972 genes, which was found to be 4.69-fold greater than the average number of genes in each genome (3191). The core genome comprised 667 genes (4.45% of the pangenome), whereas the number of unique genes varied widely, ranging from 2 to 1158 (Figure 2B). Figure 2B further illustrates that strain-specific gene content was highly uneven across the genus. While all strains shared only a relatively small core genome, some genomes contributed disproportionately large numbers of unique genes to the overall pan-genome, highlighting substantial inter-strain heterogeneity. Strain NB2B_13_BHI, which belongs to *A. hallii*, possessed the largest number of unique genes, which might be attributed to the larger genome size and higher number of CDSs (Table S1). In contrast, *A. soehngenii* CC00585 possessed the smallest number of unique genes. Furthermore, Gu et al. [38] also reported an open pan-genome and considerable genomic plasticity in *Anaerobutyricum*, consistent with our observation of an open pan-genome and large accessory/unique gene fractions.

Based on Clusters of Orthologous Groups (COG) [47] annotation, we functionally characterized the core, accessory, and unique genes of *Anaerobutyricum* using eggNOG-mapper (v2.1.6) [48] against the eggNOG 5.0 database [47], with an e-value cutoff of 1 × 10^−5^ (Figure 2C,D). The COG distributions shown in Figure 2C and the detailed functional categories in Figure 2D reveal a clear functional partitioning between conserved and variable genomic components. A high proportion (25.28%) of the pangenome consisted of genes encoding proteins with unknown functions or without homologs outside the genus (1.65% of the core genome, 19.96% of the accessory genome, 33.79% of the unique genome). The core genome was significantly enriched in genes involved in essential housekeeping functions for cellular processes, such as translation, ribosomal structure and biogenesis (J, 15.07%), amino acid transport and metabolism (E, 10.49%), and energy production and conversion (C, 9.60%). The high abundance of genes in the category “translation, ribosomal structure and biogenesis” reflects the universal role of the translational machinery, which is vital for cellular life in strains of this genus. In contrast, both the accessory and unique genomes exhibited higher proportions of genes associated with “cellular processes and signaling” and “information storage and processing” than the core genome. These genes are predominantly linked to environmental adaptation and niche specialization. Both the accessory and unique genomes were enriched in genes for replication, recombination and repair (L), transcription (K), and cell wall/membrane/envelope biogenesis (M). These functions accounted for 13.11%, 11.81%, and 6.55% of the accessory genome, and 20.86%, 11.95%, and 7.65% of the unique genome, respectively. Genes involved in replication and repair were predominantly found in the accessory and unique genomes. This suggests that strains from different isolation sources have acquired more of these adaptive genes, likely facilitating survival in fluctuating environments. Taken together, Figure 2C,D suggest that strain-level diversification in *Anaerobutyricum* is driven mainly by variation in adaptive and regulatory functions rather than by changes in essential cellular machinery.

## 4. Factors Affecting the Abundance of *Anaerobutyricum*

The abundance of *Anaerobutyricum* in the gut is influenced by multiple factors, mainly including diet, age, geographic background, and environmental conditions.

Diet is considered one of the main factors influencing the composition and metabolic functions of the gut microbiota, as changes in major nutrients or diet composition significantly affect intestinal microbes [49,50]. High-fat diets (HFDs) have been shown to induce gut dysbiosis [51]. Xiong et al. [19] reported that even short-term HFD exposure can rapidly alter the gut microbiota. In their experiment, mice were randomly divided into two groups and fed either an HFD or a regular diet for 7 days. The HFD group exhibited a significant reduction in the abundance of short-chain fatty acid (SCFA)-producing bacteria, including *A. hallii* [19]. This finding suggests that *A. hallii* is sensitive to acute changes in dietary lipid composition. In another study, feeding dairy cows a high-grain (HG) diet increased the relative abundance of *Anaerobutyricum*, accompanied by significant ileal microbial shifts and changes in bile acid metabolism and related signaling pathways [52]. In early-weaned yak calves, a 6-week feeding intervention comparing free-range management with two starter feed regimens showed that *Anaerobutyricum* was more abundant in the free-range control group, suggesting that early dietary management can influence its gut abundance [53]. Among specific dietary components, lactose may also influence the abundance of *Anaerobutyricum*. Li et al. [54] found that lactose significantly influenced gut microbial diversity when 5 g/L lactose was added to Macfarlane medium for incubating donor fecal slurries. After 48 h of fermentation, the median abundance of *Anaerobutyricum* was higher in samples incubated with 5 g/L lactose compared to the control [54]. Although *Anaerobutyricum* may not directly rely on lactose for growth, lactose supplementation was associated with an increased abundance of this genus, possibly through indirect microbial interactions. Probiotics and prebiotics can modulate the composition and function of the gut microbiota and promote the growth of beneficial bacteria, thereby helping to maintain a balanced intestinal microbial community [55]. Notably, certain probiotics, prebiotics or postbiotics can enhance the abundance of *Anaerobutyricum* by modulating gut microbial balance. For instance, supplementation with heat-treated a postbiotic preparation (heat-treated *Bifidobacterium longum* CECT 7347) in healthy adults with mild to moderate digestive symptoms was associated not only with a significant decrease in total and non-HDL cholesterol but also with an increased abundance of butyrate-producing gut bacteria, including *Anaerobutyricum* [56]. In another intervention study, administration of synbiotic fermented milk induced rapid and sustained alterations in the gut microbiota, with *Anaerobutyricum*, *Gemmiger*, *Anaerostipes*, and *Faecalibacterium* showing significant increases as early as day 3 and remaining elevated at day 28 [57]. This effect was proposed to result from lactulose-driven expansion of *Bifidobacterium*, cross-feeding interactions among gut microbes, and increased availability of amino acids and other nutrients that support the growth of butyrate-producing bacteria [57]. Similar observations have also been reported in pigs. A study investigated the effect of dietary crude protein on the pig gut microbiome and found that a low-protein diet increased the abundance of *Anaerobutyricum* in fecal samples [58]. Yi et al. [59] found that weaned pigs fed a corn/soybean-based diet and treated with raw potato starch showed a significantly increased abundance of *Anaerobutyricum*. Węsierska et al. [60] reported that supplementing the diet of broiler chickens with diatomite-bentonite mixture increased body weight gain, modulated the abundance and diversity of gut bacteria, and significantly enhanced the abundance of *A. hallii* and *A. soehngenii*. Further studies integrating multi-omics approaches are needed to elucidate how different diets influence the metabolic activity of *Anaerobutyricum* and its contribution to host health.

An increasing number of studies have indicated a profound effect of age on the gut microbiome [61,62]. Hu et al. [63] investigated the gut microbial composition in 40 pregnant women from northern China, who were divided into two groups: advanced maternal age (AMA) and younger maternal age (YMA). They reported that the relative abundance of *Anaerobutyricum* was higher in the AMA group and was significantly negatively correlated with iron levels [63]. A study of children at different stages found that the relative abundance of *A. hallii* gradually increased after six months of age and reached maximal values in the group aged 24 months and older [64]. Another study tracking the gut microbiome from infancy to five years of age found that *Anaerobutyricum* reached its peak abundance at 2 years [20]. In addition to age-related variation, early-life maternal factors may also shape *Anaerobutyricum* abundance. A longitudinal study showed that infants born to mothers with obesity were enriched in *Anaerobutyricum* at 1 month postpartum, and *A. hallii* was negatively associated with specific human milk oligosaccharides (HMOs), indicating that maternal BMI and milk glycan composition may influence its early-life establishment [65].

The composition of the gut microbiome also varies across different regions. Han et al. [66] used metagenomic analysis to characterize the gut microbiota of colorectal cancer (CRC) patients in six countries and found that some bacterial taxa were shared across all six countries, whereas the USA had the fewest bacteria in common with the other countries. The overlap between China and France was the highest, including *Anaerobutyricum* [66]. These findings suggest that geographic background may influence the distribution pattern of *Anaerobutyricum* across human populations. In addition to cross-country variation, urbanization-related lifestyle differences may also affect *Anaerobutyricum* abundance. A metagenomic comparison of rural and urban Ethiopian children showed that *Anaerobutyricum* was strongly overrepresented in the urban cohort compared with age-matched rural children, suggesting that dietary and environmental changes accompanying urbanization may shape its distribution [67].

Environmental factors such as heat stress also affect the abundance of *Anaerobutyricum*. In one study, Goel et al. [21] compared the cecal microbiota of broiler chickens between thermoneutral and heat-stress conditions, and the abundance of *Anaerobutyricum* was significantly increased under heat-stress conditions compared with thermoneutral conditions.

## 5. Physiological Functions of *Anaerobutyricum*

### 5.1. Carbohydrate Metabolism of Anaerobutyricum

**Figure 3:**
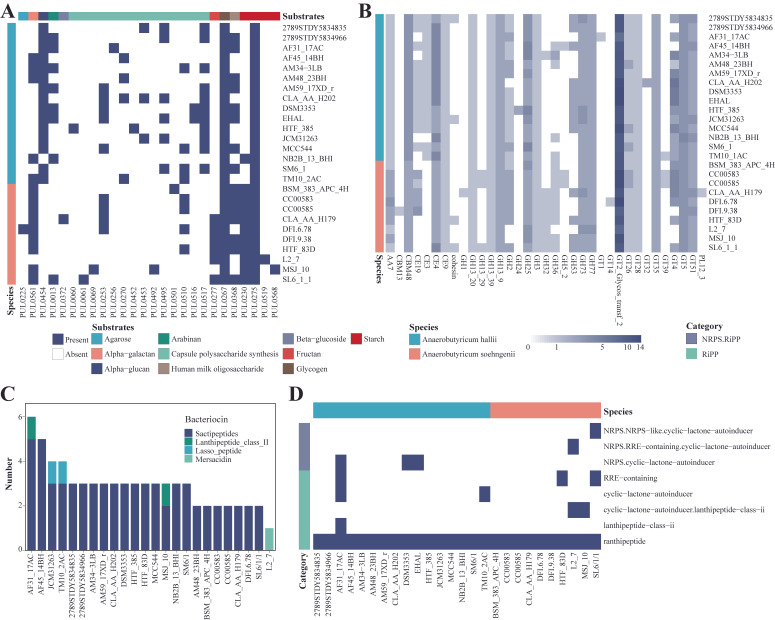
Predicted genomic traits related to carbohydrate utilization, bacteriocin production, and secondary metabolite biosynthesis in 26 *Anaerobutyricum* strains. (**A**) Distribution of predicted polysaccharide utilization loci (PULs) across strains, with predicted substrates indicated at the top. The figure highlights both conserved substrate-use potential (e.g., glycogen and starch) and marked inter-strain variation in PUL content. (**B**) Counts of carbohydrate-active enzyme (CAZyme) families in each strain. Shared GT and CE families are broadly distributed, whereas total CAZyme repertoires vary among strains, indicating functional diversity in carbohydrate metabolism. (**C**) Numbers and classes of predicted bacteriocin operons identified using BAGEL4, showing widespread but variable antimicrobial potential across the genus. (**D**) Presence/absence heatmap of predicted biosynthetic gene clusters (BGCs) in 26 strains annotated using antiSMASH (v7.0.0), illustrating strain-specific biosynthetic capacity, with ranthipeptide-related clusters among the most broadly distributed types.

Diet is considered one of the major factors influencing the colonization and abundance of *Anaerobutyricum* in the gut [19,58,59,60]. Therefore, the capacity of this genus to utilize dietary and host-derived carbohydrates is closely related to its ecological adaptation and potential physiological functions. Previous studies have shown that *Anaerobutyricum* strains can ferment a variety of sugars into SCFAs [16]. To further investigate their substrate utilization potential, we analyzed polysaccharide utilization loci (PULs) in 26 *Anaerobutyricum* genomes using dbCAN-PUL (https://bcb.unl.edu/dbCAN_PUL/ (accessed on 14 January 2026); [68]) (Figure 3A). In total, 26 PULs were predicted across these strains. These PULs were associated with the degradation of ten distinct polysaccharides, including arabinan and human milk oligosaccharides. Notably, all strains were predicted to utilize both glycogen and starch. As shown in Figure 3A, the predicted PUL repertoire varied markedly among strains, indicating substantial heterogeneity in polysaccharide utilization capacity within the genus. At the same time, glycogen- and starch-associated PULs were conserved across all strains, suggesting that these substrates may represent broadly shared carbohydrate resources for *Anaerobutyricum* in the intestinal environment. Each genome encoded more than one PUL, and the highest number of PULs per genome was nine, as observed in *A. hallii* EHAL.

Carbohydrate utilization by gut bacteria is largely determined by their repertoire of carbohydrate-active enzymes (CAZymes). CAZyme genes were searched with dbCAN2 [69] using HMMER [70] with default parameters. Across the 26 *Anaerobutyricum* genomes, 35 CAZyme domains were identified, including one auxiliary activity (AA), two carbohydrate-binding modules (CBMs), four carbohydrate esterases (CEs), one cohesin, 15 glycoside hydrolases (GHs), 11 glycosyl transferases (GTs), and one polysaccharide lyase (PL) (Figure 3B). Among these, GT2_Glycos_transf_2, GT5, and CE4 were broadly distributed and relatively abundant across all strains. Substantial intraspecific variation was observed in the CAZyme profiles of *A. soehngenii*, ranging from 18 enzymes in strain L2-7 to 25 enzymes in strain CLA_AA_H179. Figure 3B therefore indicates that the CAZyme repertoire of *Anaerobutyricum* is both conserved in broad functional capacity and variable at the strain level. The widespread occurrence of GT2_Glycos_transf_2, GT5, and CE4 suggests that these enzyme families represent common carbohydrate-processing functions in the genus, whereas the variation in total CAZyme counts highlights notable intraspecific functional diversity. Functionally, GTs are mainly involved in the biosynthesis of complex glycans, whereas GHs catalyze carbohydrate degradation [71]. These findings suggest that *Anaerobutyricum* possesses broad carbohydrate utilization potential, which may support its colonization and persistence in the intestinal environment. The ability to utilize glycogen, starch, and other polysaccharides may also contribute to SCFAs production, thereby linking dietary carbohydrate availability with the metabolic activity of *Anaerobutyricum* in the gut.

### 5.2. Bacteriocins and Secondary Metabolites of Anaerobutyricum

In addition to carbohydrate metabolism, bacteriocin production and secondary metabolite biosynthesis may represent important functional traits of *Anaerobutyricum*. Bacteriocins are antimicrobial peptides produced by bacteria and are involved in interbacterial competition and microbial community modulation [72]. For each genome, putative bacteriocin were identified using the online server Bagel 4 (http://bagel4.molgenrug.nl/index.php (accessed on 15 January 2026); [73]). Analysis of 26 *Anaerobutyricum* genomes revealed a total of 70 predicted bacteriocins, including sactipeptides, class II lanthipeptides, lasso peptides, and mersacidin-related peptides (Figure 3C). As illustrated in Figure 3C, predicted bacteriocin operons were widely distributed but unevenly represented across the analyzed genomes. The presence of multiple bacteriocin classes across strains suggests that antimicrobial competition may be a common ecological strategy in this genus, whereas variation in the number and types of operons points to strain-specific competitive capacities. These results suggest that *Anaerobutyricum* may have the potential to influence the composition of the gut microbiota through the production of antimicrobial peptides.

To further characterize the biosynthetic capacity of *Anaerobutyricum*, secondary metabolite biosynthetic gene clusters (BGCs) were annotated using antiSMASH (v7.0.0) [74]. Genome mining of the 26 *Anaerobutyricum* strains identified 39 BGCs, mainly belonging to RiPP- or NRPS/RiPP-related categories, including lanthipeptide-class-ii, NRPS.NRPS-like.cyclic-lactone-autoinducer, ranthipeptide, NRPS.cyclic-lactone-autoinducer, NRPS.RRE-containing.cyclic-lactone-autoinducer, cyclic-lactone-autoinducer.lanthipeptide-class-ii, cyclic-lactone-autoinducer, and RRE-containing (Figure 3D). Notably, ranthipeptide BGCs were generally distributed across the strains, suggesting that this type of BGC may represent a common biosynthetic feature of *Anaerobutyricum*. The similarity of these BGCs to those deposited in the MIBiG [75] repository ranged from 0 to 28%, indicating that *Anaerobutyricum* may harbor the potential to produce novel natural products. Figure 3D further shows that BGC distribution was strain dependent: some cluster types were broadly shared, whereas others were restricted to only a subset of strains. In particular, the widespread occurrence of ranthipeptide-related clusters suggests that they may represent a conserved biosynthetic trait, while the low similarity to known MIBiG entries supports the possibility that *Anaerobutyricum* encodes previously uncharacterized natural products. Based on these genomic predictions, *Anaerobutyricum* may contribute to intestinal microbial ecology not only through SCFAs production but also through antimicrobial and signaling activities mediated by bacteriocins and secondary metabolites. However, the actual expression, products, and biological activities of these predicted BGCs remain to be experimentally validated.

### 5.3. Genomic Safety Assessment of Anaerobutyricum

Although many studies have linked *Anaerobutyricum* with beneficial effects on host health, some studies have also reported associations between this genus and particular disease states [18,76]. Therefore, a genome-based safety assessment is necessary when evaluating the probiotic potential of *Anaerobutyricum*. In this study, 26 *Anaerobutyricum* genomes were screened against virulence factor and antibiotic resistance gene databases using abricate (v1.0.1; https://github.com/tseemann/abricate (accessed on 12 January 2026)) with default parameters. The virulence factor database VFDB (setB, 17 March 2025) and the antibiotic resistance gene database CARD (17 March 2025) were used. No virulence factors were detected in any of the analyzed genomes, supporting a generally favorable safety profile.

**Figure 4:**
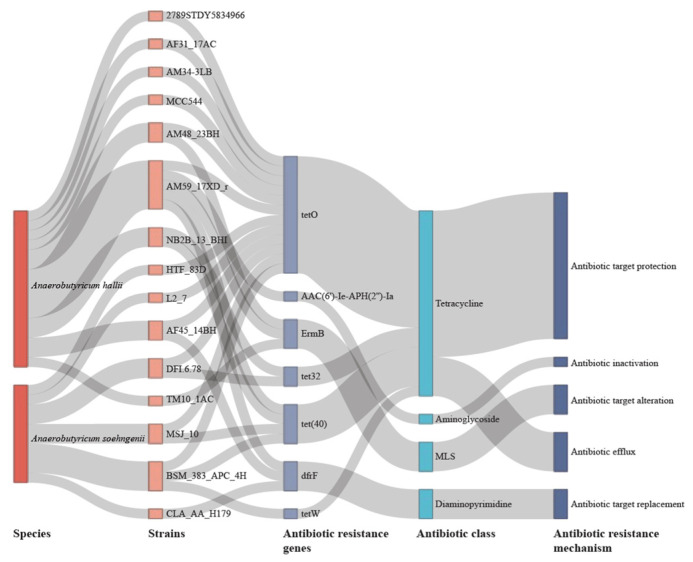
Distribution and functional classification of antibiotic resistance genes (ARGs) in *Anaerobutyricum* strains. The Sankey plot links species (first column), individual strains (second column), detected ARGs (third column), corresponding antibiotic classes (fourth column), and inferred resistance mechanisms (fifth column). The diagram shows that ARG carriage is uneven across strains, with tetracycline resistance being the most common category and several strains harboring multiple resistance determinants.

Nevertheless, antibiotic resistance genes (ARGs) were detected in 15 of the 26 genomes (57.7%) (Figure 4). As illustrated in the Sankey plot (Figure 4), ARG carriage was uneven across strains, with some genomes containing no detectable resistance determinants and others carrying multiple ARGs linked to more than one antibiotic class. Most detected ARGs converged on tetracycline resistance, which was the dominant resistance category in the dataset, whereas MLS, diaminopyrimidine, and aminoglycoside resistance were less frequently represented. These genes were associated with resistance to tetracyclines (13 strains; target protection or efflux), macrolide–lincosamide–streptogramin (MLS) antibiotics (three strains; target alteration), diaminopyrimidines (three strains; target replacement), and aminoglycosides (one strain; antibiotic inactivation). Notably, six strains exhibited multidrug resistance, defined as the presence of two or more ARGs, and strain AM59_17XD_r carried five distinct resistance determinants conferring resistance to four antibiotic classes. The presence of ARGs in *A. soehngenii* and *A. hallii* raises concerns regarding the potential horizontal gene transfer of these resistance determinants to other members of the gut microbiota. For example, although *Anaerobutyricum* has been considered a potential host of ermB, differing abundance trends between *Anaerobutyricum* and ermB suggest that this resistance gene may also be harbored by other bacterial genera [77]. Therefore, while the absence of virulence factors supports the potential safety of *Anaerobutyricum*, the mobility, expression, and transferability of ARGs should be carefully evaluated before considering specific strains for probiotic or therapeutic applications.

Recently, a comprehensive genomic study analyzed 84 *Anaerobutyricum* genomes, including both isolate genomes and metagenome-assembled genomes (MAGs), and identified integrative and conjugative elements (ICEs) in human-associated *A. hallii* and *A. soehngenii* [38]. These ICEs carry genes involved in siderophore group nonribosomal peptide biosynthesis (dltA, dhbE, mbtB) as well as antibiotic resistance genes (e.g., ermB). In contrast, our analysis based on 26 high-quality isolate genomes did not specifically examine ICEs, which may reflect differences in dataset composition (MAG-containing vs. isolate-only) and analytical scope. The presence of such ICEs suggests considerable genomic plasticity in human-associated *Anaerobutyricum* strains and further highlights the potential for horizontal dissemination of antibiotic resistance genes.

## 6. *Anaerobutyricum*-Related Diseases

Accumulating evidence suggests that changes in *Anaerobutyricum* abundance are associated with diverse pathological conditions, with both depletion and enrichment being reported depending on the disease. However, most of these findings are based on observational or association studies, and therefore do not establish whether *Anaerobutyricum* is a causal driver, a secondary responder to disease-associated ecological changes, or simply a co-varying microbial marker.

### 6.1. Diseases Negatively Associated with Anaerobutyricum

A growing number of studies have linked reduced abundance of *Anaerobutyricum* to a range of pathological conditions, particularly inflammatory, metabolic, and neurodegenerative disorders, suggesting a potentially beneficial role for this genus in host homeostasis. In multiple sclerosis, central nervous system inflammation has been associated with intestinal inflammation, elevated fecal lipocalin-2 levels, reduced microbial diversity, and depletion of beneficial bacteria, including *Anaerobutyricum* [78]. Similarly, inflammatory bowel disease (IBD), including ulcerative colitis and Crohn’s disease, has also been associated with reduced *Anaerobutyricum* abundance [79,80]. In pediatric patients with quiescent Crohn’s disease, *A. soehngenii* abundance was significantly lower in those with iron deficiency anemia than in those without anemia [81]. In ulcerative colitis, sustained remission after fecal microbiota transplantation was associated with increased abundance of *A. hallii* and enhanced butyrate-related functional capacity, including increased SCFAs production [82,83]. *A. hallii* has also been implicated in susceptibility to radiation-induced intestinal injury, although the direction and mechanism of this relationship remain unclear [84]. There is growing evidence that gut microbiota can influence the susceptibility of insulin resistance (IR) [85,86]. Vrieze et al. [87] transferred the fecal content from a healthy donor to an insulin-resistant subject by fecal microbiota transplantation (FMT), when insulin-resistant individuals received an FMT from healthy donors, their insulin sensitivity improved alongside an elevation in the abundance of *A. soehngenii* in the small intestine [87]. Although this observation is consistent with a potentially beneficial role of this bacterium in glucose metabolism, it does not by itself establish *A. soehngenii* as the causal mediator of the metabolic improvement, because FMT simultaneously alters multiple microbial and host factors. The most prevalent neurodegenerative disorders include Alzheimer’s disease (AD), and the gut microbiota can influence cognitive decline [88,89]. Analysis of fecal microbial communities in patients with subjective cognitive decline but no symptoms (SCD), mild cognitive impairment (MCI), or Alzheimer’s disease (AD) has revealed that *Anaerobutyricum* abundance is significantly higher in the SCD than in the MCI and AD groups [90]. As also observed in another study, He et al. [91] found that the abundance of *Anaerobutyricum* progressively decreased from normal controls to subjective cognitive decline and cognitive impairment. Childhood obesity is a significant health issue, wherein the gut microbiome plays a crucial role in its development [92]. Indiani et al. [85] reported a notable reduction in the abundance of *Eubacterium hallii* (now reclassified as *A. hallii* or *A. soehngenii*) in the gut microbiota of obese children. Furthermore, a study conducted on obese and insulin-resistant db/db mice demonstrated that oral administration of *A. soehngenii* enhances insulin sensitivity and energy expenditure [93]. Collectively, these findings suggest that depletion of *Anaerobutyricum* is frequently associated with compromised intestinal, metabolic, and neurological health; however, in most cases, the directionality and causality of these relationships remain unresolved.

### 6.2. Diseases Positively Associated with Anaerobutyricum

Certain gut bacteria exert beneficial effects on host health through their metabolic activities and interactions with the host, and are therefore regarded as beneficial commensals or potentially probiotic taxa. However, under different disease conditions, changes in environmental factors, microbial competition, and host immune responses may alter their ecological behavior and functional consequences, potentially contributing to inflammation or other adverse effects on host health [94,95]. Similarly, although *Anaerobutyricum* is generally regarded as a beneficial gut genus, it has also been reported to be enriched in several disease contexts, indicating a context-dependent role. For example, *A. soehngenii* was found to be enriched in the tissue microbiota of patients with triple-negative breast cancer (TNBC) compared with those with non-TNBC [18]. In infantile colic, a functional gastrointestinal disorder in early life, *A. hallii* or *A. soehngenii* was reported to be positively associated with colic-related gut microbiota dysbiosis [76]. Enrichment of *Anaerobutyricum* has also been observed in immune- and inflammation-related conditions. A metagenomic study found that *Anaerobutyricum* was more abundant in the gut microbiota of patients with rheumatoid arthritis than in healthy controls [96]. Similarly, higher abundance of *Anaerobutyricum* has been reported in coronary slow flow phenomenon, where it may form part of a disease-associated microbial signature together with elevated trimethylamine-biosynthetic taxa and selenium-related metabolic pathways [97]. Beyond the gut, altered distribution of *Anaerobutyricum* has also been observed in systemic disease. In HIV-infected individuals, blood-enriched *A. hallii* was positively associated with an expanded viral reservoir and elevated pro-inflammatory markers, suggesting that in the bloodstream this species may be linked to persistent inflammation and impaired immune restoration rather than exerting a protective commensal effect [98]. In addition, increased abundance of the *Eubacterium hallii* group has been reported in bulimia nervosa and was associated with altered tryptophan metabolism, although inconsistent findings across studies suggest that host age, disease conditions, and cohort-specific factors may influence its distribution [99,100,101]. Overall, current findings indicate that *Anaerobutyricum* is closely associated with multiple disease states, but its role appears to be highly context dependent and may vary according to host condition, disease type, and ecological niche.

It should be noted that the currently available evidence does not clearly establish the direction of causality between *Anaerobutyricum* abundance and disease states. In many studies, altered abundance of this genus has been identified under diseased conditions, but it remains unclear whether such changes contribute to disease development or instead reflect disease-driven alterations in the intestinal environment, such as shifts in substrate availability, bile acid composition, inflammation, redox status, or microbial community structure. A bidirectional relationship is also possible. On the one hand, a reduction in *Anaerobutyricum* might decrease butyrate production and disrupt metabolic cross-feeding, thereby weakening epithelial barrier function, immune regulation, and gut homeostasis. On the other hand, disease-associated changes in diet, intestinal transit, host metabolism, inflammation, or medication exposure may create ecological conditions that either suppress or enrich *Anaerobutyricum*. Therefore, changes in its abundance may represent not only a potential driver of host phenotypes but also a microbial response to disease-associated environmental perturbations. Future studies combining longitudinal sampling, strain-resolved intervention experiments, and mechanistic validation will be needed to disentangle these possibilities.

## 7. Potential Applications of *Anaerobutyricum*

### 7.1. Human Health

Compared with the relatively rich evidence linking *Anaerobutyricum* to disease-associated microbial alterations, its potential applications in human health are only beginning to emerge. *Anaerobutyricum* has been proposed as a promising next-generation probiotic candidate because of its capacity to produce butyrate, utilize lactate and acetate, and participate in microbial cross-feeding, thereby potentially contributing to intestinal metabolic homeostasis. Its specialization in lactate utilization may be especially relevant, as excessive lactate accumulation has been implicated in intestinal dysfunction [102,103]. Early translational studies provide preliminary but still limited support for its possible role in metabolic intervention. For example, increased intestinal levels of *Anaerobutyricum* were associated with improved insulin sensitivity after fecal microbiota transfer from lean donors to subjects with metabolic syndrome [87]; however, because FMT affects the gut ecosystem as a whole, this result is supportive but not sufficient to establish causality at the level of *Anaerobutyricum*. More direct evidence comes from subsequent studies showing that administration of *A. soehngenii* L2-7^T^ improved insulin sensitivity and stimulated GLP-1 secretion in experimental and early clinical settings [104]. Nevertheless, these validation data remain limited, strain-specific, and concentrated mainly in metabolic contexts. In addition, *A. hallii* has been reported to convert the dietary carcinogen PhIP into the less mutagenic metabolite PhIP-M1 through glycerol-dependent metabolism [105], suggesting a potential role in intestinal detoxification. Beyond these metabolic functions, *Anaerobutyricum* may also interact with host immunity. Notably, *A. hallii* has been identified among the most highly IgA-coated gut bacterial species [106], suggesting a close relationship with the mucosal immune system. Collectively, these findings indicate that *Anaerobutyricum* may represent a promising microbiota-targeted intervention candidate for metabolic health; however, the currently available evidence remains limited, many observations are still association based, and broader clinical application will require rigorous causal validation, strain-level mechanistic clarification, and safety assessment.

### 7.2. Swine Production

Studies on *Anaerobutyricum* in pigs remain limited, and the currently available evidence is largely association based. Given that the pig intestine is a central organ for both immunity and nutrient absorption, and that microbial-derived short-chain fatty acids can modulate these functions, gut bacteria involved in SCFA-related metabolism may have important implications for swine health and production performance [107]. Existing studies suggest that this genus may be related to several production-associated traits, including growth performance, stress responses, and reproductive performance. In weaned piglets, enrichment of *Anaerobutyricum* has been observed following probiotic or dietary interventions that were also association with improved body weight gain, feed intake, reduced diarrhea, enhanced gut barrier function, and increased SCFAs concentrations [22,108,109]. However, these findings are correlative and do not establish a direct role of *Anaerobutyricum* in these outcomes. Evidence from pathogen challenge models also suggests a possible association with host status, as *A. hallii* was more abundant in non-challenged pigs than in *Lawsonia intracellularis*-infected pigs [110], although the biological significance and causal basis of this difference remain unclear. In growing pigs, *Anaerobutyricum* was enriched in non-stressed control animals compared with pigs exposed to prolonged production-related stress [23], indicating that it may have potential as a microbial marker associated with stress status or resilience. In sows, dietary fiber supplementation increased the abundance of the *Eubacterium hallii* group, now classified as *A. hallii*, together with fecal SCFAs production and the number of live-born piglets [24], suggesting a possible association between *Anaerobutyricum*-related taxa and reproductive performance. Overall, current findings suggest that *Anaerobutyricum* may serve as a microbial indicator associated with certain production-related traits in pigs; however, direct functional evidence in swine is still lacking, host–microbe interaction mechanisms remain poorly understood, and its probiotic or nutritional intervention potential requires further strain-level, genomic, and controlled in vivo validation. At present, no study has conclusively demonstrated that *Anaerobutyricum* directly causes improvements in swine growth, intestinal health, stress resilience, or reproductive traits; therefore, its current value in pig production should be regarded as hypothesis-generating rather than functionally validated.

### 7.3. Other Animals

Although most application-oriented studies on *Anaerobutyricum* have focused on humans and pigs, emerging sequencing-based evidence suggests that this genus may also be relevant in other livestock species. In dairy cows, alterations in its abundance under high-grain feeding conditions imply a possible role in intestinal microbial adaptation and bile acid-related metabolism [52]. In poultry, *Anaerobutyricum* has been associated with growth-related and physiological traits. *Anaerobutyricum* showed positive correlations with body weight in broilers raised under commercial conditions [111], whereas dietary supplementation with a diatomite-bentonite mixture increased the abundance of *A. hallii* and *A. soehngenii* and was accompanied by improved body weight gain and altered gut microbial composition [60]. Additional evidence from native slow-growing roosters showed a positive correlation between *Anaerobutyricum* and citric acid levels [112], suggesting a possible link with intestinal metabolic activity, and heat stress has also been reported to alter its abundance in broilers, indicating potential relevance to environmental stress responses [113]. Nevertheless, these observations remain descriptive and correlation based, and the available evidence in non-swine livestock is still sparse. Therefore, the possible applications of *Anaerobutyricum* in these animals should be interpreted cautiously until further mechanistic and in vivo studies are conducted. Similarly, in other livestock species, current evidence is insufficient to distinguish whether *Anaerobutyricum* acts as a functional contributor, a secondary ecological responder, or a microbial biomarker associated with diet, physiology, or environmental stress.

## 8. Conclusions

As an emerging genus of gut microbiota, *Anaerobutyricum* plays important roles in intestinal metabolic cross-feeding, butyrate production, and host physiological regulation, and has been associated with metabolic, inflammatory, and neurodegenerative diseases as well as productive traits in pigs. However, the genus currently comprises only two validly described species, *A. hallii* and *A. soehngenii*, and most of the reported properties of this genus are still based on descriptive or association studies related to its potential probiotic functions. Therefore, the causal relationship between *Anaerobutyricum* abundance and host phenotypes remains unclear. In addition, its association with host health appears to be context dependent, as decreased abundance has been observed in some disorders whereas enrichment has been reported in others, indicating that the effects of *Anaerobutyricum* should not be overgeneralized at the genus level and that species- and strain-level differences need to be considered. Moreover, the mechanisms underlying host–*Anaerobutyricum* interactions remain poorly understood. Meanwhile, there is still insufficient evidence to support its practical application in clinical settings or animal production, particularly in livestock, where current evidence remains sparse and largely observational, and its safety requires further verification, particularly because antibiotic resistance genes have been detected in some strains despite the absence of known virulence factors in currently available genomes. Commercial production of *Anaerobutyricum*-based next-generation probiotics also remains challenging because this genus consists of strictly anaerobic bacteria that require demanding cultivation conditions and rigorous handling procedures.

Several limitations of the current evidence base should be explicitly acknowledged. First, most available studies are correlative, which limits causal inference regarding the role of *Anaerobutyricum* in host physiology and disease. Second, the currently available strain collection and genomic resources remain limited in both number and diversity, with most strains being derived from human-associated sources, thereby restricting our understanding of the ecological adaptation, host specificity, and functional diversity of this genus. Third, several functional traits discussed in this review, including predicted carbohydrate utilization capacity, bacteriocin production, and secondary metabolite biosynthesis, are based largely on genomic prediction and have not yet been sufficiently validated experimentally. Fourth, although no known virulence factors were identified in the currently available genomes, the detection of antibiotic resistance genes in some strains indicates that safety assessment remains incomplete without further evaluation of gene expression, mobility, and transferability.

To date, only limited numbers of strains have been isolated and characterized, and the available genomic information remains insufficient to fully understand the functional diversity, ecological adaptation, and host specificity of this genus. Therefore, more *Anaerobutyricum* strains should be isolated from diverse hosts, and further genomic, physiological, mechanistic, and controlled in vivo studies are needed to clarify its role in host health and evaluate its potential as a biomarker or target for microbiome-based strategies in medicine and animal agriculture. Future studies should prioritize causal validation through strain-resolved supplementation experiments, mono-colonization or gnotobiotic animal models, microbial community reconstruction, metabolite tracing, and host signaling analyses to determine whether specific *Anaerobutyricum* strains directly mediate the observed physiological effects. These efforts should be complemented by integrating comparative genomics with cultivation-based physiology, transcriptomics, metabolomics, and targeted biochemical assays to validate genome-predicted functions. From a translational perspective, key technical barriers to application also need to be addressed, including strict anaerobic cultivation, formulation stability, delivery strategies, and strain-specific safety evaluation. Overall, a combination of expanded strain discovery, mechanistic validation, and rigorous biosafety assessment will be essential for determining whether *Anaerobutyricum* can be reliably developed as a biomarker, therapeutic target, or next-generation probiotic in medicine and animal production.

## Data Availability

No new data were created or analyzed in this study. Data sharing is not applicable to this article.

## Supplementary Materials

The following supporting information can be downloaded at: https://www.mdpi.com/article/10.3390/microorganisms14061304/s1, Table S1: General genomic features of 26 strains belonging to two recognized species of *Anaerobutyricum*.
